# Molecular Basis for the Interactions of Human Thioredoxins with Their Respective Reductases

**DOI:** 10.1155/2021/6621292

**Published:** 2021-06-01

**Authors:** Md Faruq Hossain, Yana Bodnar, Calvin Klein, Clara Ortegón Salas, Elias S. J. Arnér, Manuela Gellert, Christopher Horst Lillig

**Affiliations:** ^1^Institute for Medical Biochemistry and Molecular Biology, University Medicine Greifswald, Germany; ^2^Department for Biochemistry, Karolinska Institutet, Stockholm, Sweden; ^3^Department of Selenoprotein Research, National Institute of Oncology, Budapest, Hungary

## Abstract

The mammalian cytosolic thioredoxin (Trx) system consists of Trx1 and its reductase, the NADPH-dependent seleno-enzyme TrxR1. These proteins function as electron donor for metabolic enzymes, for instance in DNA synthesis, and the redox regulation of numerous processes. In this work, we analysed the interactions between these two proteins. We proposed electrostatic complementarity as major force controlling the formation of encounter complexes between the proteins and thus the efficiency of the subsequent electron transfer reaction. If our hypothesis is valid, formation of the encounter complex should be independent of the redox reaction. In fact, we were able to confirm that also a redox inactive mutant of Trx1 lacking both active site cysteinyl residues (C32,35S) binds to TrxR1 in a similar manner and with similar kinetics as the wild-type protein. We have generated a number of mutants with alterations in electrostatic properties and characterised their interaction with TrxR1 in kinetic assays. For human Trx1 and TrxR1, complementary electrostatic surfaces within the area covered in the encounter complex appear to control the affinity of the reductase for its substrate Trx. Electrostatic compatibility was even observed in areas that do not form direct molecular interactions in the encounter complex, and our results suggest that the electrostatic complementarity in these areas influences the catalytic efficiency of the reduction. The human genome encodes ten cytosolic Trx-like or Trx domain-containing proteins. In agreement with our hypothesis, the proteins that have been characterised as TrxR1 substrates also show the highest similarity in their electrostatic properties.

## 1. Introduction

The thioredoxin (Trx) family of proteins comprises many key enzymes in redox signalling that catalyse specific reversible redox reactions, e.g., dithiol-disulphide exchange, (de-)glutathionylation, transnitrosylation, or peroxide reduction. The name giving protein, Trx, was first described in 1964 as electron donor for ribonucleotide reductase in *E*. *coli* [1, 2]; at least one functional thioredoxin system was proposed to have been encoded in the genome of LUCA, the last universal common ancestor of all live forms today [3]. Other family members include the glutaredoxins (Grx) and peroxiredoxins (Prx), often expressed in multiple isoforms in essentially all tissues, cells, and organelles [4–7]. The Trx family of proteins is defined by a common structural motif that, in its most basic form, consists of a central four-stranded *β*-sheet surrounded by three alpha helices—the thioredoxin fold [8, 9]. Most Trxs are small proteins of approximately 12 kDa size, characterised by their highly conserved CGPC active site motif located on a loop connecting sheet 1 and helix 1 (in the most basic representation of the fold) [10, 11]. Trxs catalyse reversible thiol-disulphide exchange reactions. The reduction of protein disulphides, for instance, is initiated by a nucleophilic attack of the more N-terminal active site cysteinyl residue, characterised by a particularly low pK_a_ value, on a sulphur atom of the disulphide in the target protein. This results in an intermediate mixed disulphide that, in the second reaction step, is reduced by the C-terminal cysteinyl residue, leading to the release of the reduced substrate and the formation of oxidized Trx, and for more details, see for instance [5, 12, 13]. Mammalian genomes encode approx. 20 Trxs or Trx domain-containing proteins [14]. From these, the cytosolic Trx1 (gene: TXN) is the one studied the most. Besides its two active site cysteinyl residues, cytosolic Trx1 possesses three additional structural cysteinyl residues that were implicated in regulatory function and Trx1-dimer formation [15, 16]. Trx1 does not contain a nuclear localization signal nor a signal peptide for secretion, but it was observed to translocate into the nucleus under certain conditions and also to be secreted in a nonclassic way, independent of its redox state [17–21].

Trx-oxidized active sites are reduced at the expense of NADPH by FAD-containing thioredoxin reductases (TrxR) [22]. Evolution has given rise to two classes of NADPH-dependent TrxRs: the low molecular weight (approx. 35 kDa) type and the high molecular weight (approx. 55 kDa) type [23, 24]. Both classes function as homodimers. The low molecular weight type is found in archaea, bacteria, and some eukaryota. The high molecular weight type is encoded in the genomes of higher eukaryotes including humans. Mammalian genomes encode three TrxRs: cytosolic TrxR1 (gene: TXNRD1), mitochondrial TrxR2 (gene: TXNRD2), and the thioredoxin-glutathione reductase TGR or TrxR3 (gene: TXNRD3) [25, 26]. Mammalian TrxRs are selenoproteins that form homodimers in a head-to-tail conformation. They belong to a family of pyridine-nucleotide-disulphide oxidoreductases that also includes, *e.g.*, glutathione reductase and trypanothione reductase [27]. They possess two active sites: one C-terminal GCUG motif and the N-terminal CVNVGC motif adjacent to the FAD domain. In contrast to their small molecular weight counterparts which have a high specificity for their endogenous substrate(s), these TrxRs accept a broad range of substrates [28], for human TrxR1 for instance Trx1, Grx2, and selenite; see overviews in [25, 29]. Electrons are transferred from NADPH to FAD and then to the N-terminal active site. Subsequently, the electrons are transferred to the selenocysteinyl residue containing C-terminal active site of the second protein in the dimeric TrxR and eventually to the target protein disulphides [30–32].

Numerous functions have been described for Trxs; see, *e.g*., [33]. However, the proteins cannot randomly reduce all possible protein disulphides. Instead, they show a broad but distinct substrate/target specificity, which may also be the reason for the various isoforms and Trx domain-containing proteins encoded in the genomes of higher eukaryotes. Hypotheses for the different activities and substrate specificities of Trx family proteins included differences in redox potential [34, 35], the pK_a_ values, *i.e.*, the nucleophilicity, of their more N-terminal active site cysteinyl residue [36], differences in overall dipole moments [37], and an increase in entropy as the major recognition force for Trx family protein target interactions [38].

Based on the analysis of *E*. *coli* phosphoadenylyl sulfate reductase, we have proposed that the specificity of protein-protein interactions is based mainly on two factors: first is geometric compatibility, and the second is electrostatic compatibility [39]. Based on electrostatic similarity, we have developed a mathematical approach to categorize Trx family proteins and to predict functions [14].

In this work, we propose that also the interaction of Trxs with their reductases is controlled by electrostatic compatibility. To address this hypothesis, we have analysed the interaction of the mammalian-type Trx1 with its reductase TrxR1. We have generated a number of mutants with alterations in electrostatic properties and characterised their interaction with TrxRs both *in silico* and *in vitro*; the formation and properties of the Trx1-TrxR1 enzyme-substrate complex were analysed with both wild-type proteins as well as mutants that exclude the thiol-disulphide/selenosulphide exchange reaction.

## 2. Material and Methods

### 2.1. Materials

Chemicals and enzymes were purchased at analytical grade or better from Sigma-Aldrich (St. Louis, MO, USA) unless otherwise stated.

### 2.2. Structural Analysis

Structures were acquired from the RSCB PDB Protein Data Bank (http://www.rcsb.org), and ligands and water molecules were removed using Pymol. The most representative structure of NMR ensembles was identified using UCSF Chimera [40]. The selected *in silico* mutations were inserted with the Dunbrack rotamer library [41] within the rotamer tool of Chimera; the rotamers with the highest probability were selected. The energy of the structure was minimized using Amberff14SB force field [42] after inserting a mutation in Chimera. The electrostatic calculations were performed as described before [14]. All the structures were preoriented in a way so that N-terminal active site cysteinyl residues face towards the camera perspective. These preoriented protein structures were then used to compute the electrostatic potential and the isosurfaces of the electrostatic potential. The addition of missing atoms and hydrogens, as well as the assignment of atomic charges and radii, was performed using pdb2pqr [43] applying the Amber force-field. VMD (visual molecular dynamics) [44] and APBS (Adaptive Poisson-Boltzmann Solver) [45] were used to compute the electrostatic potential in an aqueous solution containing 150 mM mobile ions, solvent dielectric constant: 78.54 at a temperature of 298.15 K. The electrostatic potential was mapped to the water accessible surface of the proteins from -4 to 4 *k*_*B*_ · *T* · *e*^−1^ represented in red and blue colours, respectively. The isosurfaces of the electrostatic potential were computed from -1 to 1 *k*_*B*_ · *T* · *e*^−1^ and also depicted in red and blue, respectively.

### 2.3. Cloning of Expression Constructs

Recombinant selenocysteine-containing rat TrxR1 (E. S. [46]) was used for all enzymatic assays, and the human TrxR U498C mutant [47] was used for all spectroscopic interaction studies, *i.e*., CD and DSF. Human Trx1 was prepared as described in [48]. *E*. *coli* Trx1 and *E*. *coli* TrxR cDNAs were amplified by PCR from human cDNA and *E*. *coli* XL1 blue, respectively, using specific primers which were designed to insert restriction sites for NdeI and BamHI (see supplementary table 1). The insert was ligated into the expression vector pET15b (Merck, Germany). All constructs and mutations were verified by sequencing (Microsynth Seqlab, Göttingen, Germany).

### 2.4. Mutagenesis

Three different groups of mutants were produced. Group I includes 10 mutants with changes in those particular residues that lead to perturbation at the immediate contact area of hTrx1 and hTrxR. Group II lists the mutants that change the electrostatic properties both in the immediate contact area and outside the contact area of hTrx1 and hTrxR. Group III includes the mutant which changes the electrostatic properties far away from the active site and immediate contact area. Mutations of the amino acid sequence and amplification of the plasmids were performed by rolling circle PCR. We generated the mutants using the indicated primers and the reversible complementary counterparts. Oligonucleotides are listed in supplementary table 1.

### 2.5. Recombinant Expression and Purification

Plasmids for recombinant expression of His-tagged Trx1 variants, *E*. *coli* TrxR, and human TrxR U498C were transformed into *E*. *coli* BL21 DE3 pRIL cells (Life Technologies, UK). Transformed cells were grown at 37°C in LB medium (Roth, Germany) with appropriate antibiotics to an optical density of 0.5 to 0.7 at 600 nm. Protein expression was induced by the addition of 0.5 mM isopropyl-1-thio-*β*-d-galactopyranoside (IPTG, Roth, Germany). The proteins were purified via immobilized metal affinity chromatography [49]. Size and purity of recombinantly expressed proteins were confirmed by SDS-PAGE using precasted TGX stain-free gels (4–20%, BioRad, Hercules, CA, USA). Pictures were taken following a five-minute UV-light activation.

### 2.6. Reduction of Proteins

The purified hTrx1 and mutant proteins were reduced with 10 mM TCEP (tris(2-carboxyethyl)phosphine) for 30 minutes and subsequently rebuffered in TE (50 mM Tris-HCl and 2 mM EDTA, pH 7.5) buffer using NAP-5 columns (GE Healthcare, UK). The rebuffered proteins were stored and kept reduced using immobilized TCEP disulphide reducing gel (Thermo Scientific, MA, USA) with a 2 : 1 ratio of sample volume to TCEP reducing gel volume. The protein and gel suspension was incubated at least 30 minutes rotating and centrifuged at 1000 × *g* for 1 minute. Protein concentration in the supernatant was determined based on molar absorptivity at 280 nm (*ε*_hTrx1_ = 6990 M^−1^·cm^−1^ and *ε*_hTrxR_ = 64290 M^−1^·cm^−1^).

### 2.7. Kinetics of Insulin Reduction by Trx

The activity of hTrx1 and different mutants was determined by a plate-based NADPH depletion assay adapted from (E. S. J. [50]). The final reaction mixtures of 200 *μ*l contained 50 mM Tris-EDTA buffer (pH 7.5), 150 *μ*M NADPH, 160 *μ*M insulin, 1.25 nM recombinant rat selenocysteine-containing TrxR and variable concentrations of Trx1, and mutant proteins (0-25 *μ*M). NADPH consumption was measured at 340 nm for 80 minutes using the Clariostar plate reader (BMG Labtech, Offenburg, Germany). The linear range of the decrease in absorption was determined for each reaction individually. Only reduced proteins were used in this assay. The specific activity of the recombinant selenocysteine-containing enzyme was approx. 100 min^−1^. This was lower than reported before [30] and likely the result of a low degree of selenocysteine incorporation into the enzyme.

### 2.8. Differential Scanning Fluorimetry

Differential scanning fluorimetry (DSF) was performed in the CFX96 real-time PCR detection system from BioRad (Hercules, CA, USA) to obtain the dissociation constant and the thermal stability of the complex as described in [51]. All the proteins were desalted and rebuffered in Tris-EDTA buffer (50 mM Tris and 1 mM EDTA, pH 7.4) after purification using NAP-5 columns (GE Healthcare, UK) prior to this assay. The final reaction mixture of 50 *μ*l contained 10 *μ*M hTrxR protein, hTrx1 with variable concentrations (from 0 to 100 *μ*M), Sypro Orange (1 : 500 diluted), and Tris-EDTA buffer. The reaction mixture was then heated with 0.3°C increments from 10.5 to 80°C. The increase in fluorescence due to binding of the dye to hydrophobic regions exposed during denaturation was recorded using the instrument's “FRET”-settings.

The melting temperatures of the thioredoxin reductase were obtained by fitting the first denaturation step (from 25.5°C to 42.6°C) with the Boltzmann fit (Eq. (1)), where FU is the measured fluorescence signal, *T*_*m*_ is the melting temperature, *T* is the temperature, and *s* is the slope.

(1) $$\begin{array}{c} \text{FU}=\frac{1}{1+\exp\left(T_{m}-T/s\right)}. \end{array}$$

The calculated melting temperatures were then plotted against the concentration of thioredoxin to obtain the EC_50_ value, which in turn can be used to calculate the dissociation constant *K*_*d*_ as described in ref. [52]. For more details, see supplementary information.

### 2.9. CD Spectroscopy

CD spectra were recorded in 300 mM NaCl and 50 mM sodium phosphate, pH 8, with the proteins hTrxR and hTrx1 at 10 and 20 *μ*M concentrations, respectively, using a Jasco J-810 spectropolarimeter from 190 nm to 240 nm at 25°C. Buffer-only spectra were subtracted. A standard sensitivity of 100 mdeg was used with a data pitch of 1 nm, 50 nm/min scanning speed, and 0.2 nm band width. In total, 10 spectra were accumulated for each sample. All the purified proteins were desalted and rebuffered in phosphate buffer (50 mM sodium phosphate and 300 mM NaCl, pH 8) using NAP-5 columns (GE Healthcare, UK) prior to CD spectroscopy.

### 2.10. Tryptophan Fluorescence

Fluorescence quenching assays were performed using a Perkin Elmer LS50B fluorimeter. The fluorescence signal was acquired at 25°C at the wavelength range from 310 to 498 nm with excitation at 296 nm. The slit width for excitation and emission was 6 and 4 nm, respectively. All purified proteins were desalted and rebuffered in Tris-EDTA buffer (50 mM Tris and 1 mM EDTA, pH 7.4) using NAP-5 columns (GE Healthcare, UK) prior to fluorescence quenching measurements.

### 2.11. All-Atom Molecular Dynamics Simulations

The crystal structures of TrxR and TrxR-Trx complex were obtained from the RCSB Protein Data Bank (PDB: 2ZZ0 and 3qfa, respectively) and then prepared for the simulations with the DockPrep tool of UCSF Chimera [40]⁠. The MD simulations were performed in Gromacs 2016.3 [53]⁠, with AMBER-99ff-ILDN force field. The proteins and complexes were solvated with TIP3P water [54]⁠ in a cubic box under periodic boundary conditions and at least 1 nm away from the edge of the box. Na^+^ and Cl^−^ ions were added to neutralize the charge of the system. An initial energy minimization was performed using the steepest descent algorithm until the system converged to 1000 kJ·mol^−1^·nm^−1^. System equilibration was performed for 100 ps at a constant number of molecules, volume, and temperature 300 K (NVT) and for a duration of 100 ps with constant number of molecules, 1 bar pressure, and temperature 300 K (NPT). The duration of each production simulation was 50-250 ns (2 fs time steps). Simulations were repeated three times. The bonded interactions of hydrogens were constrained with LINCS algorithm [55]⁠. The Parrinello-Rahman [56]⁠ method was used for pressure coupling and the modified Berendsen thermostat—velocity rescale [57]⁠ for the temperature coupling. The Particle Mesh Ewald [58]⁠ method was used for the calculation of the long-range electrostatic interactions; for the short-range interactions, Verlet cut-off scheme with 1.5 nm cut-off distance was applied, for both Coulomb- and van-der-Waals interactions. The parametrisation of FAD was done with Antechamber tool of AmberTools and ACPYPE [59]⁠ script with GAFF force field and Gasteiger charge methods. The RMSD and RMSF analyses of simulated data were performed with GROMACS-intern tools.

### 2.12. Statistical Analysis

All the numerical data are reported as mean ± SD unless otherwise stated. Statistical analyses were performed using one-way ANOVA followed by Tukey HSD test (Statistics Kingdom, Melbourne, Australia). A value of *p* < 0.05 was considered statistically significant.

### 2.13. Additional Software

All numerical calculations (spectra and kinetics) were performed and visualized using Grace (https://plasma-gate.weizmann.ac.il/Grace/). Structures were depicted using UCSF Chimera [40]. Picture panels and reaction schemes were generated using Inkscape (https://inkscape.org/).

## 3. Results

**Figure 1:**
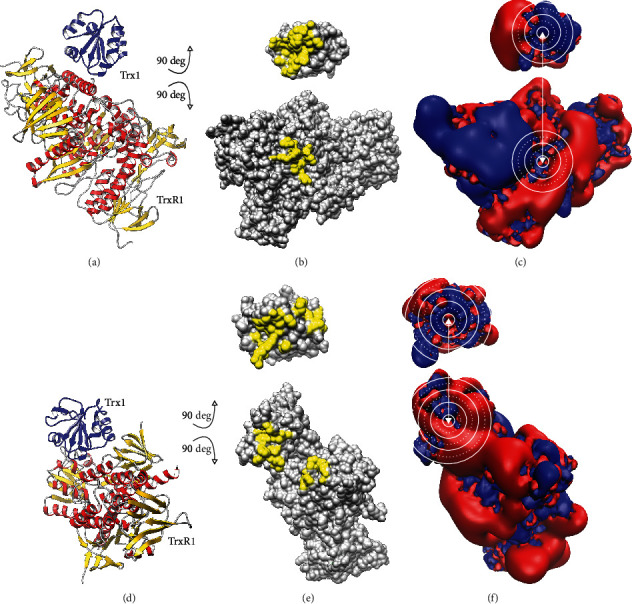
Molecular interaction surfaces of the human Trx1-TrxR1 and *E. coli* Trx1-TrxR complexes. Cartoon representation of the secondary structures of (a) human Trx1-TrxR1 complex PDB: 3qfa and (d) *E. coli* Trx1-TrxR complex PDB: 1f6m. The complex structure was opened by rotating both Trx and TrxR structures by 90° forward and backward, respectively. The contact patches with direct molecular contacts were highlighted in yellow using UCSF chimera (b, e). The isosurfaces of the electrostatic potential were depicted at ±1 *k*_*B*_ · *T* · *e*^−1^ in blue (positive) and red (negative), respectively. The active site cysteinyl residues and interaction surfaces in the immediate contact area in both proteins were encircled in white lines (c, f).

Based on previously published structures of the complexes between human Trx1 and TrxR1 [60], we have analysed the molecular interactions between the two proteins (Figure 1, supplementary Fig. 1). We have also included, for comparison, *E. coli* Trx1 and TrxR [61]. *E. coli* TrxR has a much narrower substrate specificity compared to the mammalian enzyme and cannot reduce human Trx1, while the mammalian-type TrxR1 can reduce both bacterial and mammalian Trxs (E. S. J. [50]). The first striking observation is the lower amount of molecular interactions (suppl. Fig. 1) and the smaller area of interaction, 448 versus 561 Å^2^, in the human complex (Figure 1(b)) compared to the bacterial (Figure 1(e)). We have computed the electrostatic properties of the proteins as outlined before [14] (Figures 1(c) and 1(f)). The isosurfaces of the electrostatic potentials at ±1 *k*_*B*_ · *T* · *e*^−1^ (±25.8 mV) of the interaction surfaces show an almost perfect complementarity in the immediate contact areas. Moreover, for the human complex, where only the left half of the Trx is in direct contact, as depicted in Figure 1(b), this is even true for the parts of the proteins that do not form direct molecular interactions (other than long-range electrostatic interactions), but only face each other (right side of the marked areas of the proteins in Figure 1(c)).

### 3.1. Trx and TrxR Interact Independently of the Redox Reaction and the Redox State of Trx

**Figure 2:**
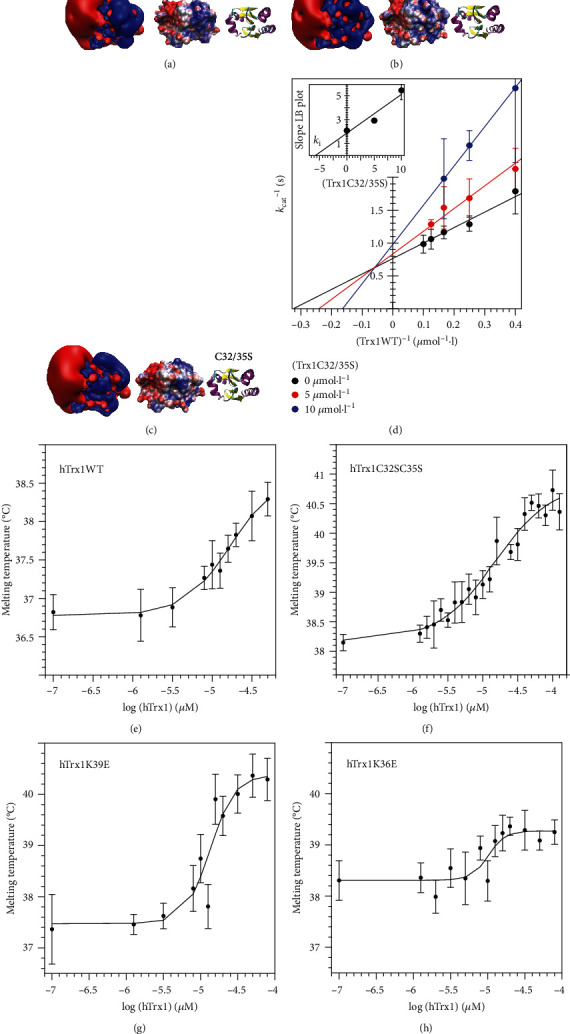
Comparison between the wild-type and double cysteinyl mutant of hTrx1 and its respective complex with hTrxR1. (a–c) Reduced and oxidized hTrx1WT compared to double cysteinyl mutant (a–c, respectively) in three different representations: (i) isosurfaces of the electrostatic potential at ±1 *k*_*B*_ · *T* · *e*^−1^ in blue (positive) and red (negative), (ii) electrostatic potential mapped on the solvent accessible surface at ±4 *k*_*B*_ · *T* · *e*^−1^, and (iii) secondary structure. (d) Lineweaver-Burk plot of inhibition kinetics measurements in the insulin reduction assay. The inset depicts the plot of the slope of the Lineweaver-Burk plots against the concentration of the redox-inactive mutant for the determination of the *k*_*i*_. This assay was performed with the recombinant selenocysteine-containing TrxR. (e–h) Hill plots depicting the log [Trx] against the melting temperature (*T*_*m*_) to determine the EC_50_ and *k*_*d*_ values of the complexes. The *T*_*m*_ values were calculated form the original melting curves fitted to the Boltzmann equation; for further details, see supplementary material. These assays were performed using the U498C mutant of human TrxR1.

We proposed that electrostatic complementarity is the major distinguishing feature that controls the specific interactions of Trx family proteins with their target proteins. The electrostatic landscape of Trx family proteins shows only minor differences between their reduced and oxidized forms (Figures 2(a) and 2(b), respectively). Interactions of the proteins, *i.e*., the formation of an encounter complex preceding the thiol or thiol-selenol disulphide exchange reactions, should therefore be independent of the subsequent redox reaction. To test this hypothesis, we have generated an inactive mutant of Trx1, in which both active site cysteinyl residues were exchanged for seryl residues (C32,35S). Figures 2(a)–2(c) depict the electrostatic characteristics of the interaction interface of reduced, oxidized, and C32,35S human Trx1 calculated from experimentally determined structures demonstrating their similarity. We have confirmed a previous report that the redox-inactive C32,35S mutant is an inhibitor of the reduction of wild-type Trx1 by TrxR1 [62]. The mutant protein inhibits the reaction by competing with the oxidized wild-type protein for binding to the reduced reductase in the insulin reduction assay (Figure 2(d)). From a secondary Lineweaver-Burk plot (inset of Figure 2(d)), the *K*_*i*_ was determined as 5.3 *μ*mol·l^−1^. Next, we analysed the complex formation by differential scanning fluorimetry (DSF, Figures 2(e)–2(h)). This method allows to determine dissociation constants by monitoring how a binding event influences the thermal stability of a protein. By fitting the data to the Boltzmann equation and plotting the log [Trx] against the *T*_*m*_ in Hill plots, we have obtained *K*_*d*_ values of 13.6 ± 1.6 and 9.8 ± 0.3 *μ*mol·l^−1^ for the wild-type and mutant proteins, respectively Table 1. For further details of this approach, we refer to the supplementary material. All together, these similarities provide further evidence that both wild-type and C32,35S Trx1 interact with TrxR in the same way. Apparently, the interaction of the two proteins does not require Trx to be oxidized, nor the thiol-disulphide/selenosulphide exchange reaction to occur.

**Figure 3:**
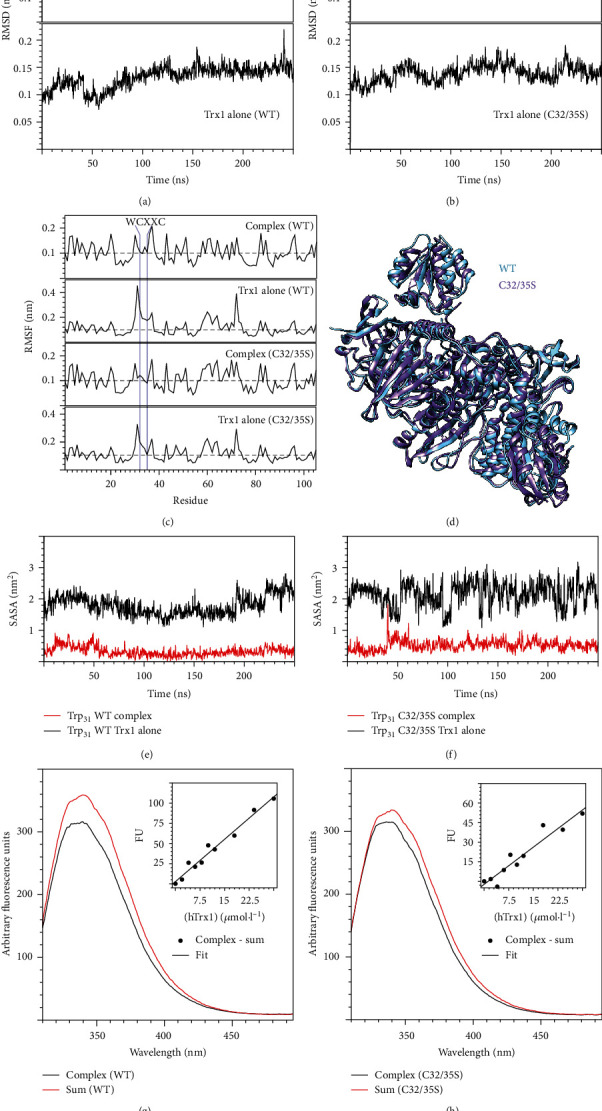
Molecular dynamics simulations of the TrxR-Trx complexes. (a, b) RMSD comparison of wild-type and C32,35S Trx alone (lower panels) and in complex with TrxR1 (top panels) over 250 ns. (c) Root-mean-square fluctuations of hTrx1 residues over 250 ns long MD simulation of wild-type and C32,35S Trx and the respective complexes with TrxR1. The active site cysteinyl residues are indicated (blue vertical lines). (d) Comparison of two representative structures from the 250 ns MD simulation of wild-type and cysteinyl double mutant complexes. The calculation of the representative structure was performed by Gromacs' clustering tool with RMSD cut-off of 0.2 nm. Superimpositioning was performed using UCSF Chimera's MatchMaker tool. (e, f) Solvent accessible surface area of W31 of hTrx1WT and hTrx1C32,35S in both complexes with hTrxR1 (red plot) and free (black plot) over 250 ns of MD simulation. (g, h) Fluorescence spectra of hTrx1-hTrxR1 U498C complexes (black) compared to the sum of the individually recorded spectra of hTrx1 and hTrxR1. The insets include the difference of the fluorescence signal between the complex- and sum spectra at 339.5 nm at different concentrations of thioredoxin.

Fritz-Wolf et al. have presented a complex structure of the mixed-disulphide intermediate snapshot between the mutant proteins Trx1 C35S and TrxR1 U498C [60]. Since this complex was enforced by using mutated proteins, which was also possible for various other permutations of point mutants of Trx1 and TrxR1 [63], we have prepared complex structures of reduced wild-type and C32,35S Trx1 with TrxR1 U498C and analysed them as well as the two Trxs alone by all-atom molecular dynamics simulations. Over the time course of 250 ns, both Trxs and both complexes behaved in a very similar way (Figures 3(a) and 3(b)). The average RMSD values of the C*α* atoms during the last 150 ns of the simulations were 1.84 and 1.93 Å for the wild-type and C32,35S complexes, respectively. The RMSD between the two most representative complexes of wild-type and mutant was 2.42 Å (complexes are shown in Figure 3(d)). The fluctuation of the side chains was also very similar, both for the free redoxins as well as for the redoxins bound to TrxR1. In general, the fluctuations of the side chains within the contact area decreased in the complexes, especially in the area of the CxxC active site (Figure 3(c), RMSF).

Essentially all Trxs contain a tryptophanyl residue immediately before the N-terminal active site cysteinyl residue. TrxR1 residue W114 is located close to the active site selenocysteinyl (in our model system cysteinyl) residue. Binding of Trx1 to the reductase should thus decrease the solvent accessibility of these two indole ring systems. Compared to the simulation runs for the redoxins alone, the solvent accessible surface area of Trx1 W31 markedly decreased in the complexes from around 2 to 0.5 Å^2^ (Figures 3(e) and 3(f)). Tryptophan indole ring systems exhibit solvatochromatic properties. In general, the fluorescence of tryptophanyl residues buried within a protein core or interaction surface is quenched due to aromatic-aromatic interactions or energy transfer to neighbouring charged groups; see for instance [64]. The decrease in solvent accessible area should thus quench the tryptophanyl fluorescence upon complex formation. We compared the changes of Trp fluorescence of both wild-type and C32,35S Trx1 upon complex formation with TrxR1 U498C. For both protein complexes, we recorded the expected fluorescence quenching (Figures 3(g) and 3(h)).

Changes in the absorption properties of Trp residues may also induce changes in near and far UV circular dichroism (CD) spectra. We have thus analysed the binding of oxidized wild-type Trx1 and the active site cysteinyl double mutant to the selenocysteinyl to cysteinyl (U498C) mutant of TrxR by CD spectroscopy (supplementary Fig. 4). We have recorded the individual spectra of both proteins and compared the sum of both spectra to the ellipticity recorded for the proteins together, *i.e*., in an equilibrium reaction of the formation and dissociation of the enzyme-substrate complex. The insets of supplementary Fig. 4A and B depict the differences in ellipticity between the spectra of the complexes and the sum. Both difference spectra do not display major differences; however, some smaller changes were found to be highly similar between the wild-type and C32,35S proteins when incubated with TrxR; these are slight increases in ellipticity at 205, 211, 217, and from 220 to 235 nm. While these changes do not prove complex formation *per se*, they provide further evidence that both the wild-type and mutant proteins interact with TrxR in a similar way.

### 3.2. Complementary Electrostatic Interaction Surfaces

**Figure 4:**
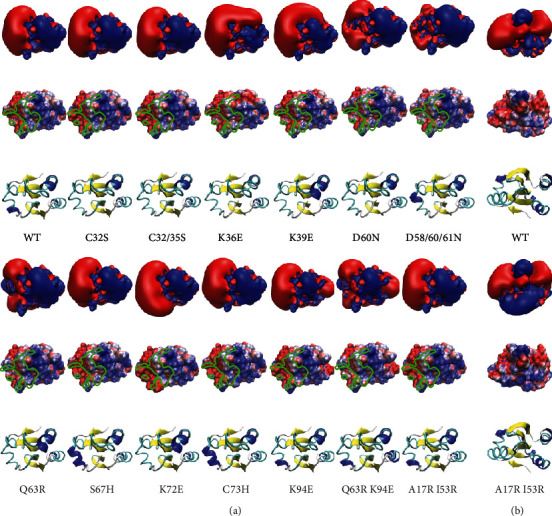
Overview of hTrx1 mutants in different representations. The first row shows the isosurfaces of the electrostatic potential at ±1 *k*_*B*_ · *T* · *e*^−1^ in blue (positive) and red (negative), respectively. The second row shows the electrostatic potential mapped to the water accessible surface at ±4 *k*_*B*_ · *T* · *e*^−1^. The third row displays the cartoon representation of the secondary structure of wild-type and mutant Trx1s (a, b). The immediate contact areas of interaction surfaces were circled in green in the second row of both panels (a). All the structures are oriented so that the N-terminal active site cysteinyl residues face towards the camera perspective (a) or rotated by 180° (b).

**Figure 5:**
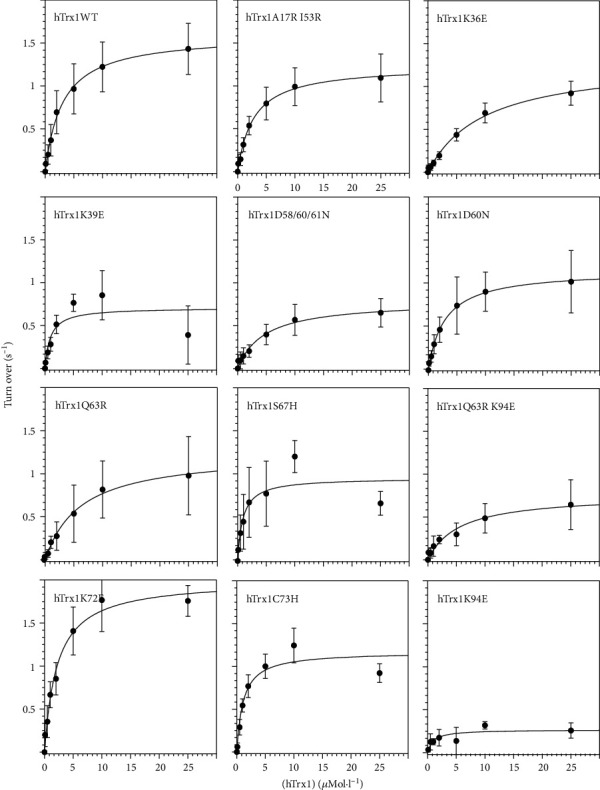
Kinetics of the reduction of the Trx1 mutants by TrxR. Michaelis-Menten plots for the proteins analysed. For details, *e.g.*, on statistics and number of independent experiments, see Table 1 of the main text. All data are shown as mean ± SD. The curves are the nonlinear curve fittings to the Michaelis-Menten equation from which the kinetic constants were obtained. These assays were performed with the recombinant selenocysteine-containing TrxR.

To analyse the importance of complementary electrostatic interaction surfaces for the mammalian Trx1-TrxR redox couple, we have generated a number of Trx1 mutants that change the electrostatic potential (Figure 4): first, within the immediate contact area but, with one exception (K72), not involved in direct molecular interactions (for these, please see suppl. Fig. 2A-B); second, outside the immediate contact area but on the surface that faces the reductase; and third, one mutant with profound changes on the opposite side of the interaction surface (Figures 4(a) and 4(b)). Following recombinant expression in *E. coli* and purification applying immobilized metal affinity chromatography, we have confirmed the folding and stability of all mutants using differential scanning fluorimetry (suppl. Fig 3). Using a coupled assay with the reduction of insulin, to keep the Trx in the assay mix oxidized, we have analysed the reaction kinetics of recombinant selenocysteine-containing TrxR1 with these mutants as substrates, as summarized in Table 2 and Figure 5.

The mutations within or partly within the interaction surface (K36E; D60N; D58,60,61N; Q63R; and Q63R,K94E) mostly resulted in a significantly reduced catalytic efficiency of TrxR. This decreased efficiency was the result of an increase in *K*_*M*_, *i.e.*, a reduced affinity of TrxR for these proteins as substrates. The exception to this was the S67H mutation with only subtle changes to the electrostatic potential close to the N-terminal active site thiol (Figure 4). For this mutant, the catalytic efficiency did not change significantly. However, this was the result of both a significant decrease in *K*_*M*_ and *k*_cat_. Two mutants with changes in their electrostatic characteristics outside the immediate contact surface but still facing TrxR (K39E and K94E) were analysed. For K39E, the catalytic efficiency did not change significantly. Again, this was the result of both a significant decrease in *K*_*M*_ and *k*_cat_. The K94E mutant was the biggest surprise in our analysis. The changes (positive to negative) introduced here (see Figure 4) affected an area approx. 10 Å away from the active site thiol and the immediate contact area. Nevertheless, TrxR displayed the lowest catalytic efficiency with this mutant as a result of both an increase in *K*_*M*_ and a drop in *k*_cat_. The double mutant Q63R,K94E that combines changes within and outside the area forming molecular interactions with TrxR (other than long-range electrostatic interactions) caused an increase in *K*_*M*_ and a decrease in *k*_cat_, resulting in a catalytic efficiency comparable to those of the single mutants (Table 2). Changes in the electrostatic potential on the side opposite to the interaction surface of Trx1 (A17R,I53R) led to a small, but not significant, drop in *k*_cat_ and did not change the affinity of TrxR1 for this protein compared to the wild-type.

We have also tested some of our human Trx1 mutants, *i.e.*, those that were more similar to *E. coli* Trx1 (Q63R,K94E; S67H; K72E; and K94E, see Figure 4 and suppl. Fig. 6) as substrate for the *E. coli* reductase. As the wild-type hTrx1, these failed to interact with the bacterial enzyme (suppl. Fig. 5).

### 3.3. Human Cytosolic Trxs and Trx Domains

**Figure 6:**
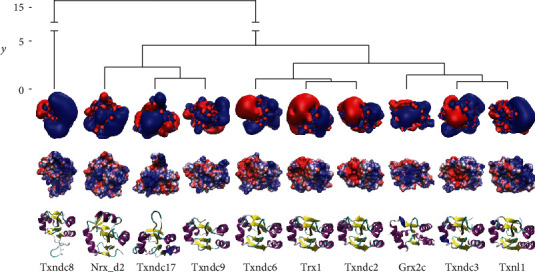
Electrostatic similarity of human cytosolic thioredoxins and thioredoxin domain-containing proteins. The tree of electrostatic similarity (on top) was prepared as described in [14]. The first row shows the isosurfaces of the electrostatic potential at ±1 *k*_*B*_ · *T* · *e*^−1^ in blue (positive) and red (negative), respectively. The second row shows the electrostatic potential mapped to the water accessible surface at ±4 *k*_*B*_ · *T* · *e*^−1^. The third row displays the cartoon representation of the secondary structure of wild-type and mutant Trxs. The orientation of the structures is the same as in Figures 1 and 4(a).

The human genome encodes at least ten cytosolic Trxs or Trx domain-containing proteins, *i.e.*, Trx1, Txndc2, 3, 6, 8, 9, and 17, Txnl1, and Nrx. To our knowledge, only Trx1, Txndc2, Txndc17 (also known as TRP14), and Txnl1 have been experimentally confirmed as substrates of TrxR1 [65–67]. Grx2, that is expressed in cytosolic and mitochondrial isoforms [68], has also been characterised as substrate for TrxR1 [69]. Nrx (nucleoredoxin) has been suggested as TrxR1 interaction partner [70].  Figure 6 summarizes the electrostatic similarity of these proteins. The highest similarity in the electrostatic properties was observed in the proteins that were characterised as TrxR1 substrates before. Interestingly, Trxndc17 differs from the other redoxins significantly; however, it does show complementarity when rotated 180°, suggesting that this could reflect a unique mode of interaction with TrxR1 (suppl. Fig. 6).

## 4. Discussion

The results presented here confirm that the binding of Trx1 to TrxR1 is independent of a subsequent redox reaction between the proteins. Trx1 does not need to be in the oxidized disulphide form to bind to TrxR1. The redox-inactive Trx1 C32,35S, presumably always in a conformation that reflects reduced wild-type protein, binds to both reduced and oxidized TrxR1. The redox reaction-independent binding of Trx to its reductase has been reported before. In 1994, Oblong and coworkers first reported that human Trx1 C32,35S is a competitive inhibitor of the reduction of wild-type Trx1 by TrxR with a *K*_*i*_ value of 6.7 *μ*mol·l^−1^ [62], and the *K*_*i*_ of 5.3 *μ*mol·l^−1^ estimated in this study is in good agreement with this. This value is close to the *K*_*M*_ of the enzyme for wild type Trx1 (2.4 *μ*mol·l^−1^), demonstrating that TrxR1 has similar affinities for both the wild-type and the redox-inactive mutant. Oblong and coworkers also reported detectable changes in CD spectra when wild-type and C32,C35S were bound to TrxR [62]. Subsequently, the C32,35S mutant has been characterised as a dominant negative mutant of the Trx system when overexpressed *in vivo*. For instance, in 1996, Gallegos et al. analysed the effect of Trx overexpression on the phenotype of breast cancer cells. They reported that wild-type Trx expression increased cell proliferation, but the expression of the C32,35S mutant inhibited cell growth and reversed the transformed phenotype of the cells. Xenografted into immunodeficient mice, wild-type Trx1 expression increased tumour formation, while expression of the redox-inactive mutant inhibited tumour formation [71]. Yamamoto *et al.* reported in 2003 that transgenic cardiac-specific overexpression of the C32,35S mutant of Trx1 diminished the endogenous activity of Trx [72]. Oh *et al.* reported in 2004 the up- and downregulation of matrix metalloprotease 2 activities upon expression of wild-type and C32,35S Trx1, respectively, in human dermal fibroblasts [73]. More recently, Das reported on the effects of transgenic overexpression of both wild-type and C32,35S Trx1 in lung tissue. Wild-type Trx1 increased the resistance to hyperoxia-induced lung injury and increased the levels of reduced Trx in the lung. Overexpression of the redox-inactive mutant, however, decreased Trx activity and even increased the degree of oxidation of endogenous wild-type Trx in the tissue [74]. Taken together, these results demonstrate that the binding of Trx1 to TrxR is independent of the subsequent thiol-disulphide/selenosulphide exchange reaction and does not require Trx to be in the oxidized disulphide form.

Based on the analysis of the interaction of *E. coli* Trx and phosphoadenylyl sulfate (PAPS) reductase, Palde and Carroll suggested that Trxs recognize the oxidized form of its target proteins with higher selectivity compared to their reduced counterparts and that an increase in entropy may be a major recognition force for their interaction [38]. Based on this observation, the authors proposed a universal entropy-driven mechanism for thioredoxin-target recognition. It should, however, be mentioned that the Trx/PAPS redox couple may not be the most representative redox couple to study the importance of the individual redox states, because reduction and oxidation of PAPS reductase require extensive conformational changes of the protein [75–78]. For the Trx-TrxR redox couple discussed here, our results—as well as all the before mentioned evidence—imply that the redox state does not seem to have a major influence on the recognition and formation of a complex between the two proteins.

Peng *et al.* studied the reactivity of TrxA, TrxP, and TrxQ from *Staphylococcus aureus* with persulphidated pyruvate kinase as a model substrate. The three redoxins displayed small differences in substrate specificity that were also discussed to be the result of electrostatic differences in the area surrounding the N-terminal active site thiol [79]. We have previously proposed and provided evidence that the binding of Trx family proteins to their interaction partners and likely all protein-protein interactions in aqueous solution require geometrically compatible surfaces and, that given, are controlled by complementary electrostatic surfaces [14, 39, 80]. Here, we have engineered mutants of Trx1 with changes in their electrostatic potential landscape within and outside the contact patch with TrxR. In summary, the inversion of positive or negative potentials in areas that fell within the immediate contact area with TrxR1 decreased the affinity of the enzyme for its substrate without affecting *k*_cat_. The subtle changes introduced with the S67H mutation close to the cysteinyl residue attacked by the selenol of TrxR in the reaction cycle decreased both *K*_*M*_ and *k*_cat_. The effects of the reversals in the electrostatic potential outside the immediate contact surface but still facing TrxR depend on the positions. While the more negative potential “north” of the N-terminal active site cysteinyl residue (see Figures 1(c) and 2) did not change the catalytic efficiency of TrxR, and the positive to negative inversion 10 Å “east” of the active side (K94E) caused the largest decrease in catalytic efficiency. The positive potential of K94 faces complementary negative potentials on TrxR; however, the oxygen atoms of the hydroxyl and carboxy groups responsible for this negative potential are in 18-22 Å distance of the *ε*-amino group of K94. These distances exclude direct long-range electrostatic interactions as a potential explanation. Under the given conditions, these should only be significant until a distance of around 6 Å.

For our study, the tryptophanyl residues W31 and W114 of Trx1 and TrxR1, respectively, proved to be valuable for the analysis of the interaction between these two proteins. W114 of TrxR1 is an unusually reactive residue. If not part of the interaction surface with Trx, W114 is a solvent accessible residue susceptible to oxidation. It was suggested that this may serve regulatory functions; it may also serve as an electron relay communicating with the FAD moiety. When oxidized, it facilitates oligomerisation of TrxR1 into tetramers that were also found in a crystal structure of TrxR1 [63]. Moreover, oxidatively modified W114 was suggested to contribute to covalently bonded, but not disulphide-linked, dimers between TRP14 and TrxR1 in cells [81].

Human and *E. coli* TrxR exhibit significant differences in their substrate specificities. While the human reductase accepts Trxs from different species and various low molecular weight compounds, *E. coli* TrxR is basically restricted to its endogenous Trx substrates, and for an overview, see (E. S. J. [50]). To some degree, especially for the reduction of low molecular weight compounds, this may be due to the higher reactivity of the selenolate active site in the human enzyme compared to the thiolate in its bacterial counterpart. Our study here provides a hypothesis for the distant specificities of the bacterial and mammalian reductases for Trxs. The *E. coli* enzyme requires significant more molecular interactions (see Figure 1 and suppl. Figs. 2C and D). The contact area is larger and concave. This requires a considerably higher degree of geometrical complementarity. Moreover, its electrostatic properties are more delicate and less binary compared to the human. The human genome encodes at least ten cytosolic Trxs or Trx domain-containing proteins, *i.e.*, Trx1, Txndc2, 3, 6, 8, 9, and 17, Txnl1, and Nrx. Trx1, Txndc2, and Txnl1 have been confirmed as substrates of TrxR1 [66, 67]. Txndc17 (also known as TRP14) was reported to be reduced efficiently by TrxR1 but not by TrxR2 ([65, 82], 14). This is in contrast to Trx1 that can be reduced by both reductases. Grx2, that is expressed in cytosolic and mitochondrial isoforms [68], has also been characterised as substrate for TrxR1 [69]. Nrx (nucleoredoxin) has been suggested as TrxR1 interaction partner [70].  Figure 6 summarizes the electrostatic similarity of these proteins. In agreement with our hypothesis, the proteins that have been characterised as TrxR1 substrates also show the highest similarity in their electrostatic properties. The only exception to this is Txndc17 that is clearly more distant. In fact, the electrostatic properties of its interaction surface are basically the opposite to the other functional redoxins (see Figure 6 and suppl. Fig. 6). Since this ought to block any fruitful interaction with TrxRs, we propose that Txndc17 must interact in a different way with TrxR1 compared to the other proteins. If the orientation of the interaction surface of Txndc17 is rotated by 180°, a fruitful interaction may become possible (see suppl. Fig. 6). The lack of activity with TrxR2 may then be the result of geometric constrains that inhibit these alternative interactions, since the attack of the selenolate on the redoxin disulphide has to occur in an 180° angle in line with the disulphide. A high geometric and electrostatic complementarity is required for the binding of a Trx to *E. coli* TrxR. For the human TrxR1, the lower number of direct molecular interactions and its more protruding active site (see Figure 1(a)) may contribute to its ability to bind and reduce a greater variety of Trxs. With this decreased importance of geometric complementarity, the electrostatic compatibility may be the primary factor controlling the enzyme affinity for different redoxins as well as the efficiency of catalysis (see Figure 4 and Table 2). That is why the human enzyme also reduces *E. coli* Trx1 that only displays limited complementarity (see Figure 1 and suppl. Fig. 6), albeit with 14-fold lower affinity and 15.4-fold lower catalytic efficiency (E. S. J. [50]).

Conclusions

Our study provides new insights into the molecular interactions between human Trx1 and its reductase TrxR1. We confirmed that the transient protein-protein interactions, *i.e.*, the formation of an encounter complex between the proteins, are independent of the subsequent redox reaction. The proteins must have an inherent affinity for each other in the area of the thiol-disulphide/selenosulphide exchange reaction controlling fruitful collisions in solution. The velocity of the reaction is too fast to be the result of random collisions between the proteins only. The only molecular forces that act in considerable distance in solution are electrostatic attraction and repulsion. For human Trx1 and TrxR1 electrostatic complementarity within the area covered in the encounter complex, it appears to control the affinity of the reductase for Trx, whereas electrostatic complementarity in areas outside this contact area can have a large influence on the catalytic efficiency.

## Figures and Tables

**Table 1:** Dissociation constants and binding energy determined for the interactions between Trx1 and TrxR1.

hTrx1 mutants	*n*	*K*_*d*_	Δ*G*	ΔΔ*G*
		*μ*mol·l^−1^	kJ·mol^−1^	kJ·mol^−1^
WT	12	13.60 ± 1.63	−27.79 ± 0.29	0
C32,35S	12	9.80 ± 0.31	−28.59 ± 0.2	-0.79
K36E	12	5.28 ± 0.08	−30.12 ± 0.03	-2.33
K39E	12	9.68 ± 1.25	−28.64 ± 0.31	-0.85

**Table 2:** Kinetic analysis of the TrxR1 catalysed reduction of Trx1 variants in an insulin reduction-coupled assay.

Categories	hTrx1 mutants	*n*	*K*_*M*_	*k*_cat_	Catalytic efficiency	
			*μ*mol·l^−1^	s^−1^	%	mol·l^−1^·s^−1^
Wild-type	WT	43	2.43 ± 0.54	1.55 ± 0.42	100.00%	6.38·10^−7^

Inside or partly within the contact surface	K36E	13	9.82 ± 1.83^∗^	1.33 ± 0.28	21.23%	1.35·10^−7^
	D60N	16	3.26 ± 1.76	1.19 ± 0.42	57.23%	3.65·10^−7^
	D58,60,61N	17	5.83 ± 2.05^∗^	0.93 ± 0.16	25.01%	1.6·10^−7^
	Q63R	15	6.37 ± 1.57^∗^	1.25 ± 0.57	30.76%	1.96·10^−7^
	S67H	18	1.36 ± 0.75^∗^	1.03 ± 0.31^∗^	118.73%	7.57·10^−7^
	K72E	18	2.22 ± 0.86	1.99 ± 0.39^∗^	140.53%	8.96·10^−7^
	C73H	16	1.24 ± 0.36^∗^	1.22 ± 0.17	154.25%	9.84·10^−7^

Mixed type	Q63R,K94E	15	7.09 ± 1.88^∗^	0.88 ± 0.3^∗^	19.46%	1.24·10^−7^

Outside the contact surface but facing TrxR	K39E	18	1.45 ± 0.43^∗^	0.86 ± 0.14^∗^	92.98%	5.93·10^−7^
	Q63R,K94E	15	7.09 ± 1.88^∗^	0.88 ± 0.3^∗^	19.46%	1.24·10^−7^
	K94E	6	3.78 ± 1.56^∗^	0.45 ± 0.16^∗^	18.66%	1.19·10^−7^

Opposite side	A17R,I53R	18	2.73 ± 0.49	1.24 ± 0.32	71.21%	4.54·10^−7^

## Data Availability

All data not already contained with the manuscript will be made available by the corresponding author upon reasonable request.

## Supplementary Materials

Supplementary Materials The following supplementary material is available separately: Supp. Table 1: oligonucleotides used in this study; further details on the *K*_*d*_ determination from the differential scanning fluorimetry data; Suppl. Fig. 1: examples for the determination of *T*_*m*_ values; Suppl. Fig. 2: molecular interactions between Trxs and their reductase; Suppl. Fig. 3: thermal stability of the Trx mutants analysed in this study; Suppl. Fig. 4: circular dichroism spectra of hTrx1-hTrxR1 complexes; Suppl. Fig. 5: human Trx mutants as potential substrates of E. coli TrxR; Suppl. Fig. 6: comparison of the electrostatic features of human TrxR1, TrxR2, E. coli TrxR, human Trx1, and Trp14, as well as E. coli Trx1; additional references.
