# Supplementary material for: Molecular Basis for the Interactions of Human Thioredoxins with Their Respective Reductases
Source: Oxid Med Cell Longev. 2021 Jun 1;2021:6621292. doi: 10.1155/2021/6621292 (PMC8189816; doi:10.1155/2021/6621292)
Supplement: Supplementary Materials — The following supplementary material is available separately: Supp. Table 1: oligonucleotides used in this study; further details on the Kd determination from the differential scanning fluorimetry data; Suppl. Fig. 1: examples for the determination of Tm values; Suppl. Fig. 2: molecular interactions between Trxs and their reductase; Suppl. Fig. 3: thermal stability of the Trx mutants analysed in this study; Suppl. Fig. 4: circular dichroism spectra of hTrx1-hTrxR1 complexes; Suppl. Fig. 5: human Trx mutants as potential substrates of E. coli TrxR; Suppl. Fig. 6: comparison of the electrostatic features of human TrxR1, TrxR2, E. coli TrxR, human Trx1, and Trp14, as well as E. coli Trx1; additional references. [file 6621292.f1.pdf]

Supplementary material to:

Molecular basis for the interactions of human thioredoxins with their respective reductases

Md Faruq Hossain[1,§], Yana Bodnar[1,§], Calvin Klein[1], Clara Ortegón Salas[1], Elias S.J. Arnér[2,3], Manuela Gellert[1], and Christopher Horst Lillig[1*]

From the Institute for Medical Biochemistry and Molecular Biology, University Medicine Greifswald, Germany (1), the Department for Biochemistry, Karolinska Institutet, Stockholm, Sweden (2) and the Department of Selenoprotein Research, National Institute of Oncology, Budapest, Hungary (3).

[§] These authors contributed equally to this work

* Address for correspondence: Institute for Medical Biochemistry and Molecular Biology, University Medicine Greifswald, Ferdinand-Sauerbruch-Straße, DE-17475 Greifswald, Germany, Tel: +49 3834 865407, Fax: +49 3834 865402, E-mail: horst@lillig.de

Supplementary Tables

Suppl. Table 1 – Oligonucleotides used in this study.

Cloning of E.coli Trx-system		
Target		Primers
E.c.Trx1	forward	5'-CACACACATATGAGCGATAAAATTATTCACCTGACTGACGACAG-3'
	reverse	3'-CACACAGGATCCTTACGCCAGGTTAGCGTCGAGGAAC-5'
E.c.TrxR1	forward	5'-CACACACATATGGGCACGACCAAACACAGTAAACTGCTTATCCTG -3'
	reverse	3'-CACACAGGATCCTTATTTTGCGTCAGCTAAACCATCGAGGTAGCG TTCC-5'
Mutagenesis of hTrx1		
Mutant		Primers
C32S	forward	5'- CCACGTGGTCTGGGCCTTGCAAAATGATCAAGCC-3'
	reverse	3'-GGCTTGATCATTTTGCAAGGCCCAGACCACGTGG-5'
C32/35S	forward	5'- CTTCTCAGCCACGTGGTCTGGGCCTTCCAAAATGATCAAGCC-3'
	reverse	3'-GGCTTGATCATTTTGGAAGGCCCAGACCACGTGGCTGAGAAG-5'
K36E	forward	5'-GTGGGCCTTGCGAAATGATCAAGC-3'
	reverse	3'-GCTTGATCATTTCGCAAGGCCCAC-5'
K39E	forward	5'- CCTTGCAAAATGATCGAGCCTTTCTTTC-3'
	reverse	3'-GAAAGAAAGGCTCGATCATTTTGCAAGG-5'
A17R	forward	5'-GCTTTTCAGGAAGCCTTGGACCGTGCAGGTGATAAACTTGTAG-3'
	reverse	3'-CTACAAGTTTATCACCTGCACGGTCCAAGGCTTCCTGAAAAGC-5'
I53R	forward	5'-GTATTCCAACGTGCGCTTCCTTGAAGTAGATGTGG-3',
	reverse	3'-CCACATCTACTTCAAGGAAGCGCACGTTGGAATAC-5'
D60N	forward	5'-CCTTGAAGTAGATGTGAATGACTGTCAGGATG-3'
	reverse	3'-CATCCTGACAGTCATTCACATCTACTTCAAGG-5'
D58/60/61N	forward	5'-CCTTGAAGTAAATGTGAATAACTGTCAGGATGTTGC-3'
	reverse	3'-GCAACATCCTGACAGTTATTCACATTTACTTCAAGG-5'
Q63R	forward	5'-CACACTCTGAAGCAACATCACGACAGTCATCCACATCTACTTC-3'
	reverse	3'-GAAGTAGATGTGGATGACTGTCGTGATGTTGCTTCAGAGTGTG-5'
S67H	forward	5'-CAGGATGTTGCTCACGAGTGTGAAGTC-3'

	reverse	3'-GACTTCACACTCGTGAGCAACATCCTG-5'
K72E	forward	5'- TCAGAGTGTGAAGTCGAGTGCATGCCAACATTC -3'
	reverse	3'-GAATGTTGGCATGCACTCGACTTCACACTCTGA-5'
C73H	forward	5'-GAGTGTGAAGTCAAACACATGCCAACATTCC-3'
	reverse	3'-GGAATGTTGGCATGTGTTTGACTTCACACTC-5'
K94E	forward	5'-GGTGGCTTCAAGCTTTTCCTCATTGGCTCCAGAAAATTCACCC-3'
	reverse	3'-GGGTGAATTTTCTGGAGCCAATGAGGAAAAGCTTGAAGCCACC-5'

Further details on the K_d determination from the differential scanning fluorimetry data

To obtain the melting temperature of the thioredoxin reductase-thioredoxin complex, the melting temperature of the first melting step T_m (from 25.5 to 42.6°C) was evaluated. For that, the measured fluorescence intensity of SYPRO Orange (FU) was plotted against the temperature (T) and then fitted to the Boltzmann equation (eq. (1)).

$$FU = a_1 + \frac{a_1 - a_0}{1 + \exp\left(\frac{T_m - T}{s}\right)} \tag{1}$$

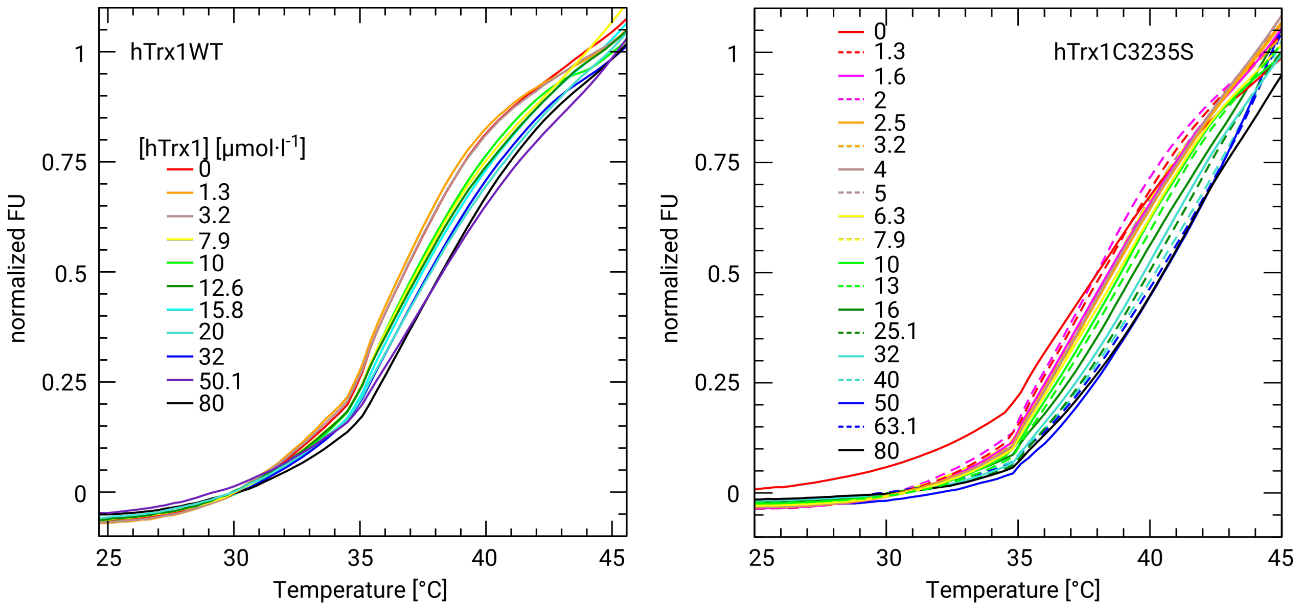

Suppl. Fig. 1 Examples for the determination of T_m values. Temperature against fluorescence units of SYPRO Orange for wild-type and the redox-inactive C32,35S mutant of human Trx1.

The melting temperatures could then be plotted against the concentration of thioredoxin. This sigmoidal curve was fitted to Hill's equation (eq. (2)) in order to obtain the EC_{50} value of the complex formation (For the Hill plots see Fig. 2 of the main text).

$$T_m = c_0 + \frac{c_1 - c_0}{1 + 10^{n \cdot \log_{10}(EC_{50}) - \log_{10}([hTrx1])}} \tag{2}$$

Next, the EC_{50} could be used to calculate the dissociation constant of the complex as described in (Bai et al. 2019). For that, the fraction of the unfolded TrxR at 25°C in absence of Trx was

calculated. Under the assumption that the normalized fluorescence intensity is corresponding to the fraction of the unfolded protein, equation (3) could be used.

$$f_{u0}=\frac{1}{1+\exp(\frac{T_m-25°C}{s})} \tag{3}$$

With that, the dissociation constant of the TrxR-Trx complex can be calculated as described in equation (4).

$$K_d=(1-f_{u0})\cdot(E_{50}-\frac{[hTrxR1]}{2}) \tag{4}$$

Supplementary Figure 2 – molecular interactions between Trxs and their reductase

Ligplot (Laskowski and Swindells 2011) analysis of the interactions between Trx and TrxR in the complexes of the human (pdb 3qfa) **A-B** and *E.coli* Trx1-TrxR complex (pdb 1f6) **C-D**. Both structures included interactions between the homo-dimeric TrxRs and two Trxs molecules, hence two interactions sets each.

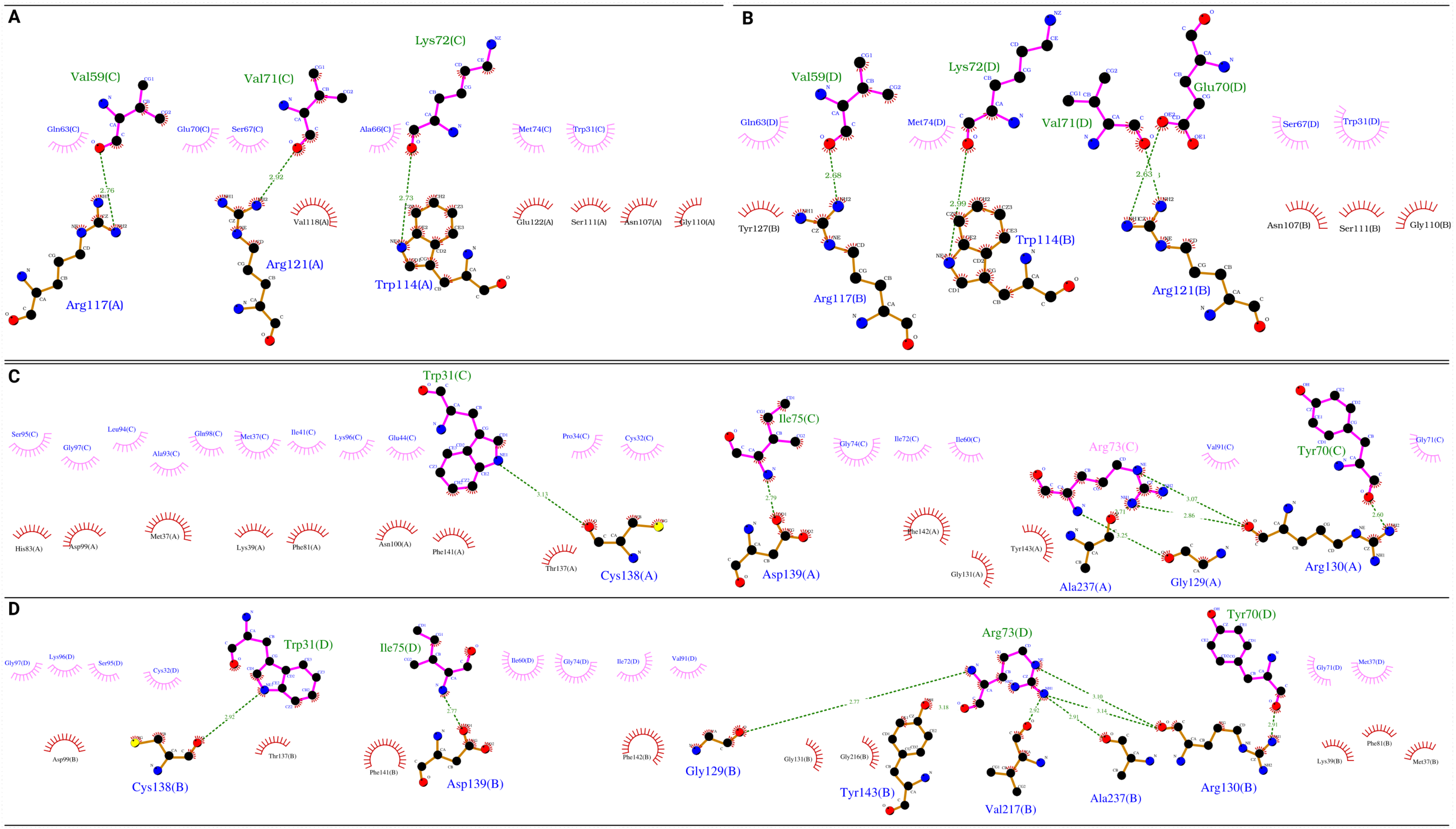
A
Val59(C)
Val71(C)
Lys72(C)
Arg117(A)
Arg121(A)
Trp114(A)
Gln63(C)
Glu70(C)
Ser67(C)
Ala66(C)
Met74(C)
Trp31(C)
Val118(A)
Glu122(A)
Ser111(A)
Asn107(A)
Gly110(A)
B
Val59(D)
Lys72(D)
Val71(D)
Glu70(D)
Arg117(B)
Trp114(B)
Arg121(B)
Gln63(D)
Met74(D)
Ser67(D)
Trp31(D)
Tyr127(B)
Asn107(B)
Ser111(B)
Gly110(B)
C
Trp31(C)
Ile75(C)
Arg73(C)
Tyr70(C)
Cys138(A)
Asp139(A)
Ala237(A)
Gly129(A)
Arg130(A)
D
Trp31(D)
Ile75(D)
Arg73(D)
Tyr70(D)
Cys138(B)
Asp139(B)
Gly129(B)
Tyr143(B)
Val217(B)
Ala237(B)
Arg130(B)

Supplementary figure 3 – thermal stability of the Trx mutants analysed in this study.

The thermal stability was analysed using a differential scanning fluorimetry approach, also know as thermofluor assay (Ericsson et al. 2006). All proteins analysed at 50 µmol·l^{-1} appeared to be stable and in a native state under the conditions analysed in this study. The red lines are the mathematical fits to the Boltzmann equation (see main text).

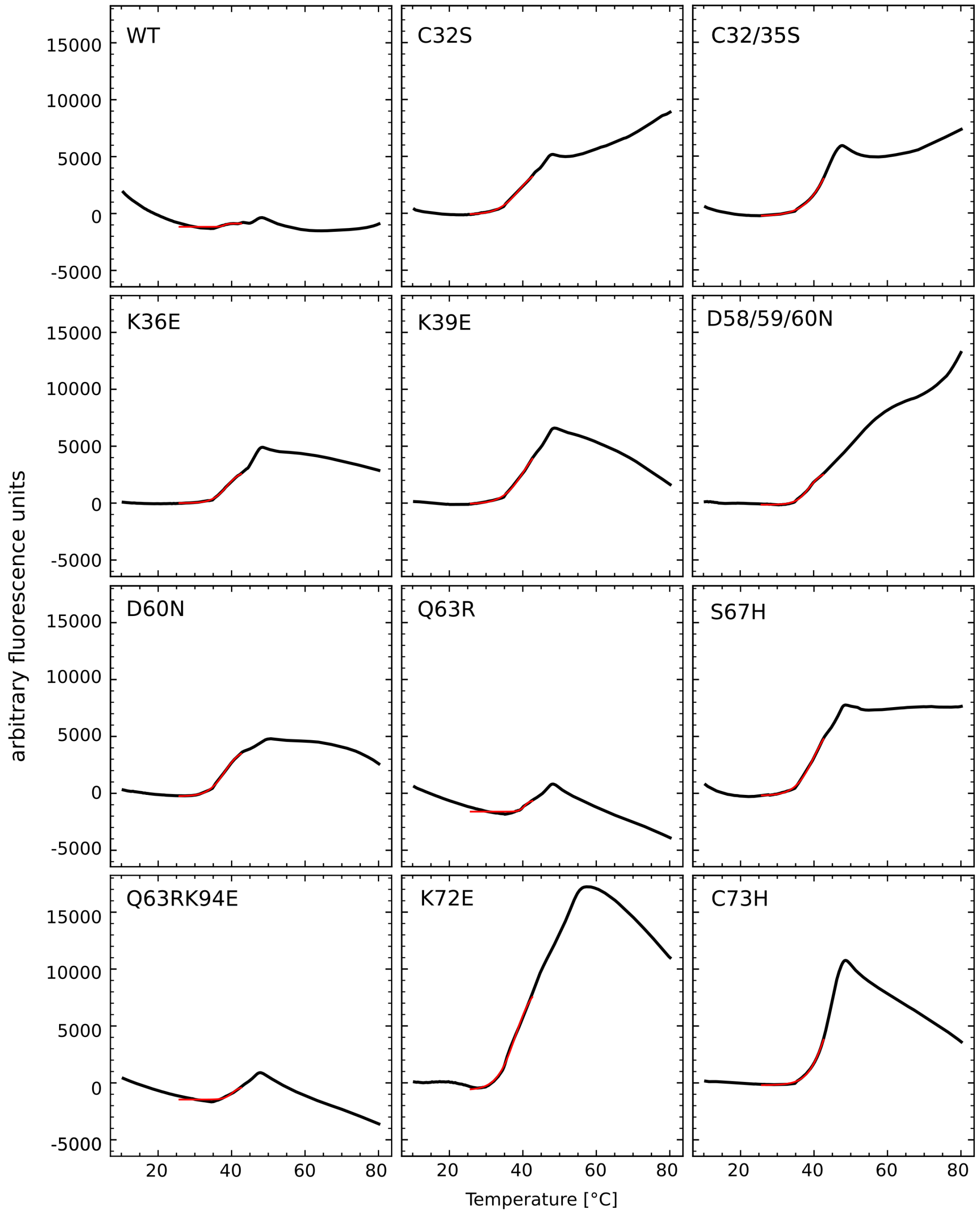

Supplementary figure 4 - Circular dichroism spectra of hTrx1-hTrxR1 complexes

Circular dichroism spectra of hTrx1-hTrxR1 complexes (black) compared to the sum between individual spectra (red); the inset plots show the difference between these two plots. This assay was performed using the C498U mutant of human TrxR.

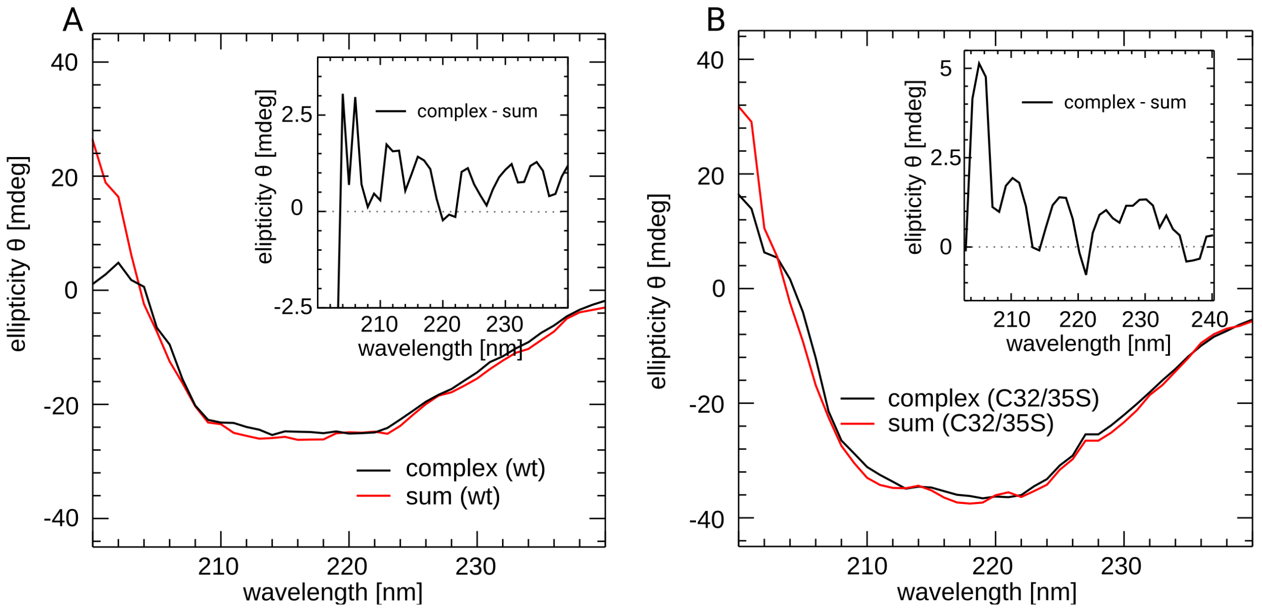

Supplementary figure 5 – human Trx mutants as potential substrates of *E. coli* TrxR.

Michaelis-Menten plot for the proteins analysed. For details, *e.g.* on statistics and number of independent experiments, see table 1 of the main text. All data are shown as mean with standard deviation. The curves are the non-liner curve fittings to the Michaelis-Menten equation from which the kinetic constants were obtained.

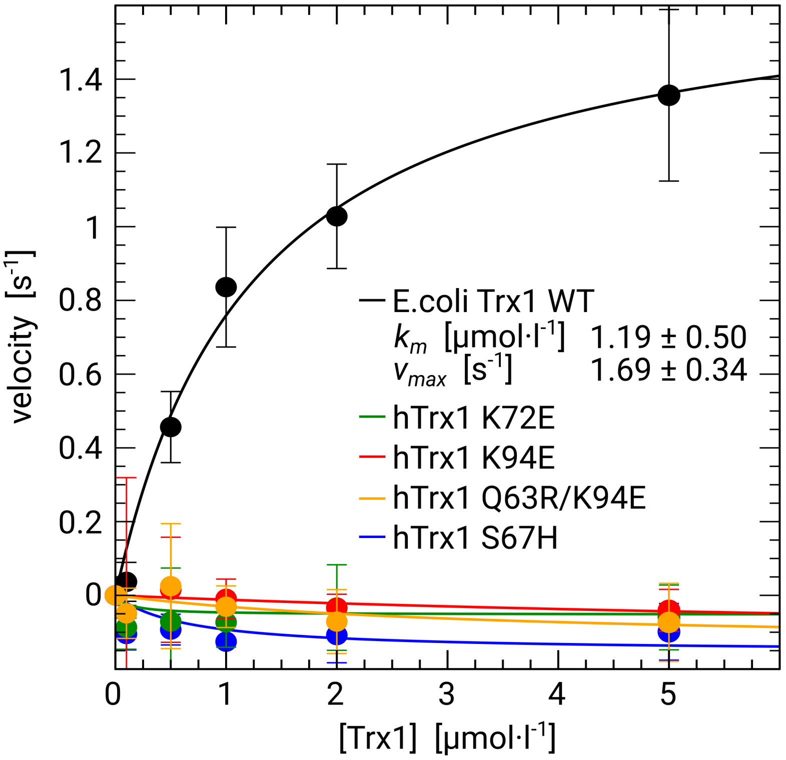

Supplementary figure 6 – Comparison of the electrostatic features of human TrxR1, TrxR2, E. coli TrxR, human Trx1 and Trp14, as well as E. coli Trx1.

The isosurfaces of the electrostatic potential were depicted +/- 1 $k_B \cdot T \cdot e^{-1}$ blue (positive) and red (negative), respectively. The active site cysteinyl residues and interaction surfaces in the immediate contact area in both proteins were encircled in white line.

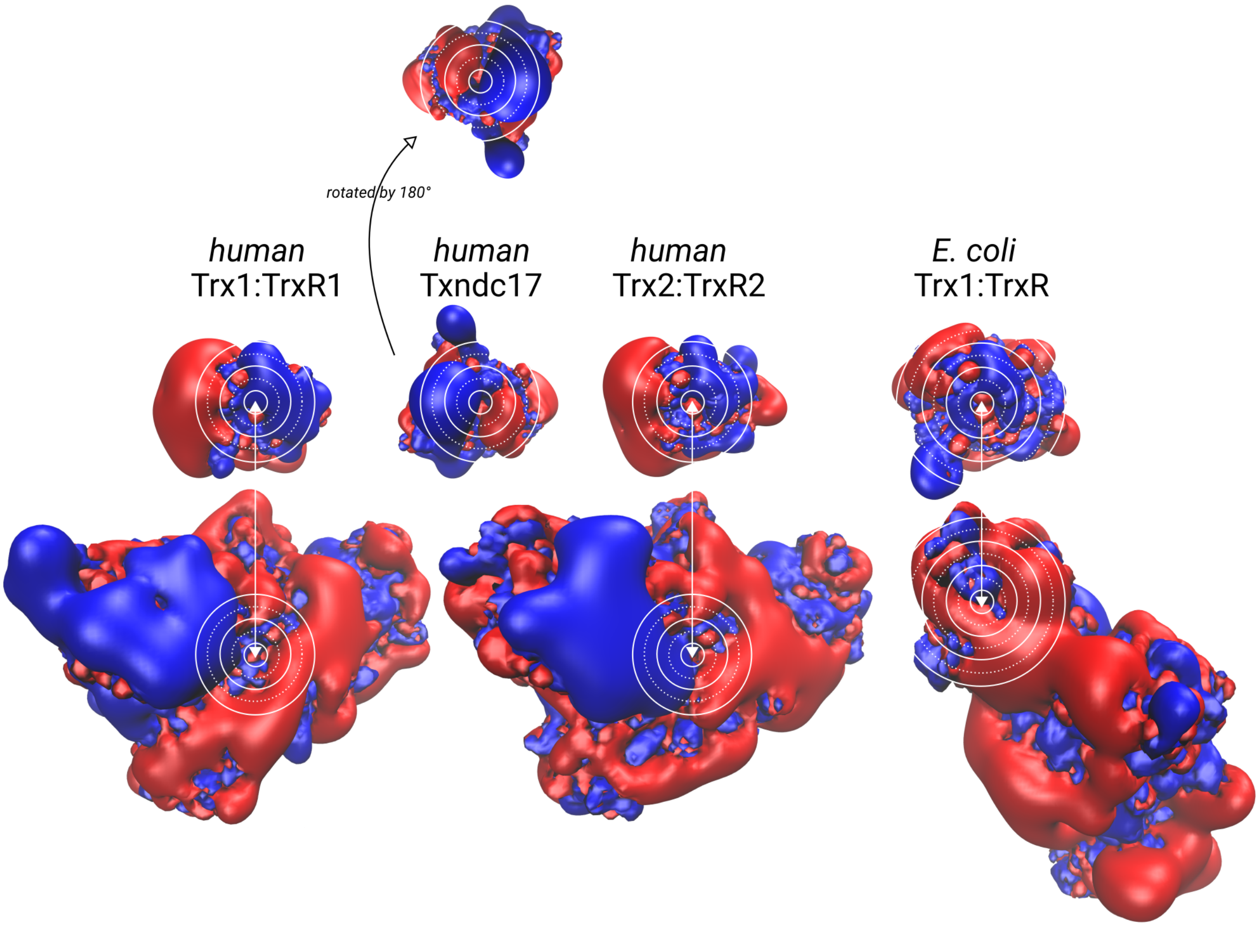

Additional references

Bai, Nan, Heinrich Roder, Alex Dickson, and John Karanicolas. 2019. “Isothermal Analysis of ThermoFluor Data Can Readily Provide Quantitative Binding Affinities.” *Scientific Reports* 9 (1): 1–15. https://doi.org/10.1038/s41598-018-37072-x.

Ericsson, Ulrika B, B Martin Hallberg, George T Detitta, Niek Dekker, and Pär Nordlund. 2006. “Thermofluor-Based High-Throughput Stability Optimization of Proteins for Structural Studies.” *Analytical Biochemistry* 357 (2): 289–98. https://doi.org/10.1016/j.ab.2006.07.027.

Laskowski, Roman A., and Mark B. Swindells. 2011. “LigPlot+: Multiple Ligand-Protein Interaction Diagrams for Drug Discovery.” *Journal of Chemical Information and Modeling* 51 (10): 2778–86. https://doi.org/10.1021/ci200227u.
